# Clonal Expansions of CD8^+^ T Cells with IL-10 Secreting Capacity Occur during Chronic *Mycobacterium tuberculosis* Infection

**DOI:** 10.1371/journal.pone.0058612

**Published:** 2013-03-05

**Authors:** Joshua C. Cyktor, Bridget Carruthers, Gillian L. Beamer, Joanne Turner

**Affiliations:** 1 Department of Microbial Infection and Immunity, The Ohio State University, Columbus, Ohio, United States of America; 2 Department of Veterinary Biosciences, The Ohio State University, Columbus, Ohio, United States of America; 3 Center for Microbial Interface Biology, The Ohio State University, Columbus, Ohio, United States of America; University of Palermo, Italy

## Abstract

The exact role of CD8^+^ T cells during *Mycobacterium tuberculosis* (*Mtb*) infection has been heavily debated, yet it is generally accepted that CD8^+^ T cells contribute to protection against *Mtb*. In this study, however, we show that the *Mtb*-susceptible CBA/J mouse strain accumulates large numbers of CD8^+^ T cells in the lung as infection progresses, and that these cells display a dysfunctional and immunosuppressive phenotype (PD-1^+^, Tim-3^+^, CD122^+^). CD8^+^ T cell expansions from the lungs of *Mtb*-infected CBA/J mice were also capable of secreting the immunosuppressive cytokine interleukin-10 (IL-10), although *in vivo* CD8^+^ T cell depletion did not significantly alter *Mtb* burden. Further analysis revealed that pulmonary CD8^+^ T cells from *Mtb*-infected CBA/J mice were clonally expanded, preferentially expressing T cell receptor (TcR) Vβ chain 8 (8.2, 8.3) or Vβ 14. Although Vβ8^+^ CD8^+^ T cells were responsible for the majority of IL-10 production, *in vivo* depletion of Vβ8^+^ did not significantly change the outcome of *Mtb* infection, which we hypothesize was a consequence of their dual IL-10/IFN-γ secreting profiles. Our data demonstrate that IL-10-secreting CD8^+^ T cells can arise during chronic *Mtb* infection, although the significance of this T cell population in tuberculosis pathogenesis remains unclear.

## Introduction

The factors that are responsible for the reactivation of latent *Mtb* infection are not well understood, but likely involve contributions from both the host and the pathogen. To appreciate the role that the host immune system plays in *Mtb* reactivation, we used relatively resistant (C57BL/6) or susceptible (CBA/J) mice, whose susceptibility phenotype is most apparent during late stages of infection, to represent differences in the natural progression of TB between different human populations. CBA/J mice have low numbers of antigen-specific CD4 T cells that produce relatively small amounts of IFN-γ [1]–[4]. CBA/J mice also have elevated amounts of IL-10 during *Mtb* infection [5], [6], contributing to their increased susceptibility to infection. However, the importance of CD8^+^ T cells during *Mtb* infection in this mouse strain remains unclear.

CD8^+^ T cells are an important component of the protective immune response to *Mtb*, as defined by studies showing that mice deficient in CD8^+^ T cells had impaired control of *Mtb* infection [7]–[10]. Although there is no consensus on the specific requirement for CD8^+^ T cells during *Mtb* infection, CD8^+^ T cells can contribute to *Mtb* control by secretion of IFN-γ [11], [12] and cytotoxic lysis of host cells [13], [14], yet their ability to maintain maximal effector function is dependent on CD4^+^ T cells [15]–[17]. Studies have also reported that CD8^+^ T cells are most important during latent *Mtb* infection in mice, and that CD8^+^ T cell depletion early after infection had little effect on disease outcome [18]. Conversely, other studies suggest that CD8^+^ T cells are dispensable during *Mtb* infection [19]–[21].

In chronic viral infection models, CD8^+^ T cells can become dysfunctional after chronic antigenic stimulation, characterized by a lack of functional or proliferative capability, secretion of IL-10 [22]–[24] and surface expression of inhibitory molecules, such as programmed cell death-1 (PD-1) and T cell immunoglobulin and mucin protein-3 (Tim-3) [25], [26]. PD-1 has classically been used as a marker of T cell exhaustion in viral infection and in cancer [27]–[30], while other studies have found that cells expressing Tim-3 are dysfunctional and lack regulation [31], [32], and that coexpression of PD-1 and Tim-3 leads to extensive dysfunction of CD8^+^ T cells [33]. Furthermore, CD8^+^ T cells expressing both PD-1 and CD122 (the β subunit of the IL-2 receptor) have been shown to have suppressive qualities and secrete IL-10 [34]. We, and others, have previously demonstrated that *Mtb* susceptibility in CBA/J mice is mediated by excessive pulmonary IL-10 during infection [1], [2], [5], [35], [36], yet the underlying mechanism remains unclear. Although numerous cell types are capable of producing IL-10, studies have previously shown that IL-10-producing T cells can actively suppress the immune response in TB patients [37], supporting an investigation into the IL-10-producing properties of CD8^+^ T cells during *Mtb* infection in CBA/J mice.

In this study we show that *Mtb*-susceptible CBA/J mice accumulated large numbers of CD8^+^ T cells in their lungs as *Mtb* infection progressed that could not be fully accounted for by an expansion of IFN-γ-producing CD8^+^ T cells. CD8^+^ T cell expansions expressed the inhibitory molecules PD-1, Tim-3, and/or CD122, and were capable of secreting IL-10. CD8^+^ T cells from CBA/J mice also preferentially expressed TcR Vβ8 and Vβ14, severely limiting the diversity of the CD8^+^ T cell repertoire. Although Vβ8 CD8^+^ T cells could secrete IL-10, *in vivo* depletion of this specific T cell clonal population during chronic infection did not overtly change the *Mtb* burden in the lungs in the timeframe tested, although the amount of IL-10 in the lung was reduced indicating some biological impact of depletion. Comparing mouse strains that are relatively resistant and susceptible to *Mtb* has enabled us to uncover a previously unappreciated role for CD8^+^ T cells in *Mtb* susceptibility, and links the poor T cell function previously described by us [4], [6], [36] with increased production of IL-10 in the CBA/J mouse strain.

## Materials and Methods

### Ethics Statement

This study was carried out in strict accordance with the recommendations in the Guide for the Care and Use of Laboratory Animals of the National Institutes of Health. The protocol was approved by the Institutional Animal Care and Use Committee of The Ohio State University.

### Mice

Specific pathogen-free, age/sex-matched CBA/J wild-type (National Cancer Institute, NIH, Frederick, MD), C57BL/6 wild-type (Jackson Laboratories, Bar Harbor, Maine), or CBA/J IL-10^−/−^ mice were maintained in ventilated cages inside a biosafety level 3 (BSL3) facility and provided with sterile food and water *ad libitum*. To generate CBA/J IL-10^−/−^ mice, CBA/J mice (Jackson laboratories, Bar Harbor, Maine) were crossed with C57BL/6 IL-10^−/−^ mice (Jackson) for eight generations. At each cross progeny mice were ear-punched and DNA was screened for the presence of a neomycin cassette at the *il10* gene locus. IL-10^+/−^ mice were selected for further breeding. At the eighth generation, heterozygotes were crossed and IL-10-deficient homozygote CBA/J mice were selected. A homozygous breeder colony of CBA/J IL-10^−/−^ mice was maintained thereafter. All protocols were approved by The Ohio State University's Institutional Laboratory Animal Care and Use Committee.

### *Mtb* Infection and Colony Forming Unit Enumeration

*Mtb* Erdman (ATCC 35801) was obtained from the American Type Culture Collection (Manassas, VA). Stocks were grown in Proskauer-Beck liquid medium containing 0.05% Tween 80 to mid-log phase and frozen in 1 mL aliquots at −80°C. Mice were infected with *Mtb* Erdman using an inhalation exposure system (Glas-Col) calibrated to deliver 50–100 CFU to the lungs of each mouse, as previously described [38]. At specific time points post *Mtb* infection mice were sacrificed and lungs were aseptically removed into sterile saline. Organs were homogenized and serial dilutions plated onto 7H11 agar supplemented with OADC as previously described [4]. Plates were incubated at 37°C for 21 days in order to enumerate bacterial colonies and calculate the bacterial burden.

### Cell Isolation

Mice were euthanized by CO_2_ asphyxiation and lungs perfused with cold phosphate buffered saline containing 50 Units/mL of heparin through the right ventricle of the heart. Lungs from individual mice were mechanically disrupted using a GentleMACS dissociator (Miltenyi Biotec, Boston, MA) followed by collagenase A (type XI) (0.7 mg/mL, Sigma) and type IV bovine pancreatic DNAse (30 µg/mL, Sigma) digestion at 37°C for 30 minutes in GentleMACS C-tubes. Lung cell suspensions were passed through a 70 µm nylon cell screen and residual erythrocytes were lysed with Gey's solution. Viable cells were determined by trypan blue exclusion.

### Cell Purification

Single lung cell suspensions were adhered to sterile tissue culture dishes for 1 hr at 37°C. Non-adherent cells were washed and removed from the plates. CD4^+^ and CD8^+^ T cells were obtained from the non-adherent cell fraction by magnetic cell separation (BD IMAG anti-CD4^+^ particles GK1.5, anti-CD8^+^ particles 53–6.7) and either placed directly into TRIzol reagent (Invitrogen, Grand Island, NY), homogenized, and frozen at −80°C or used for culture as described below. CD8^+^Vβ8^+^ T cells were obtained from the CD4^neg^ fraction after treatment with anti-CD4 magnetic beads (BD), then stained with PE anti-Vβ8 (eBioscience) and purified using anti-PE magnetic particles (BD). Purity of all CD4^+^ and CD8^+^ T cell populations was determined to be greater than 90% for all experiments by flow cytometry using an LSRII flow cytometer (BD Biosciences, San Jose, CA).

### Cytokine Assays

For ELISpot, bone marrow-derived dendritic cells (BMDCs) were obtained from the tibiae and femora of age and sex matched non-infected wild-type or IL-10^−/−^ C57BL/6 or CBA/J mice. Cells were differentiated into dendritic cells using complete DMEM supplemented with 10% conditioned media derived from GM-EL4 cells, a GM-CSF-producing clone kindly provided by Arthur A. Hurwitz (NCI). 2×10^6^ bone marrow cells were plated at 37°C in 1 ml of GM-EL4 conditioned media in sterile 24-well tissue culture plates. GM-EL4 conditioned media was replaced on days 2, 4 and 6. 3×10^4^ BMDCs were infected overnight with *Mtb* Erdman at an MOI of 1∶1 then fixed in 2% paraformaldehyde (CBA/J IL-10^−/−^ BMDCs were used unfixed). Infected BMDCs were cultured with 2×10^5^ CD8^+^ T cells or CD8^neg^ T cells for 72 hr at 37°C in media containing either tissue culture media alone or 10 µg/mL anti-CD3 (145-2C11) and 1 µg/mL anti-CD28 (37.51). ELISpot reagents were obtained from eBioscience Ready-Set-GO! Spot-forming units (SFU) were enumerated with an ELISpot plate counter (C.T.L.).

For ELISA, 1×10^5^ purified pulmonary CD8^+^ T cells were cultured with 10 µg/mL anti-CD3 (145-2C11) and 1 µg/mL anti-CD28 (37.51) for 72 hr at 37°C with 5% CO_2_. After incubation, plates were frozen at −80°C until all timepoints were completed. ELISA antibodies and standards were obtained from BD Biosciences and processed as previously described [38]. Colormetric reactions were read on a SpectraMax plate reader (Molecular Devices, Sunnyvale, CA).

### Flow Cytometry

Isolated lung cells or MLN were suspended in deficient RPMI (Mediatech, Manassas, VA) supplemented with 0.1% sodium azide (Sigma-Aldrich). Surface targets were detected as previously described. Specific antibodies and isotype controls were purchased from BD Biosciences: PerCP-Cy5.5 anti-CD3ε (145-2C11), allophycocyanin-Cy7 anti-CD4^+^ (GK1.5), PE-Cy7 anti-CD8^+^ (53–6.7), PerCP-Cy5.5 anti-CD8^+^ (53–6.7), PE-Cy7 anti-IFN-γ (XMG1.2), PE anti-PD-1 (J43), FITC anti-CD122 (TM-Beta 1), and FITC Vβ screening kit. PE anti-Tim-3 (HAVCR2) antibody was purchased from eBiosciences. Cytokine levels were determined according to the manufacturer's instructions for intracellular cytokine staining (Cytofix/Cytoperm fixation/permeabilization solution kit with BD GolgiStop, BD Biosciences), following a 4 hr incubation with 1 µg/mL anti-CD3 (145-2C11) and 0.1 µg/mL anti-CD28 (37.51). Samples were read using an LSRII flow cytometer and analyzed with FACSDiva software (BD Biosciences).

### Cell Depletion

Anti-CD8^+^ (53.6.72) depletion antibody (BioXCell) or whole rat IgG2a (2A3) (BioXCell), were diluted to 2.5 mg/ml in PBS and stored at –80°C. Thawed stocks were maintained at 4°C for up to 1 month. Anti-Vβ8 (F23.1) antibody-secreting cell line [39] was kindly provided by Dr. Michael Bevan. Antibody administration was modified based on Silva et al. [40] as follows: at day 90 post-infection, 0.5 mg of anti-CD8^+^ (53.6.72), anti-Vβ8 (F23.1), or control antibody was injected into the peritoneal cavity of each mouse, followed by 0.5 mg at weekly intervals thereafter until the designated experimental time points. At the initiation of antibody treatment CBA/J mice consistently harbor 6.59± SE 0.12 log_10_ *Mtb* CFU [4].

### Statistics

Statistical analysis performed using GraphPad Prism software for the Students *t* test per individual time point of each graph. Any comparisons between timepoints of the same experiment utilize a two-way analysis of variance test with Bonferonni post-tests for multiple comparisons. * p<0.05, ** p<0.01, *** p<0.001.

## Results

### CD8^+^ T cells accumulate in the lungs of CBA/J mice as *Mtb* infection progresses

**Figure 1:**
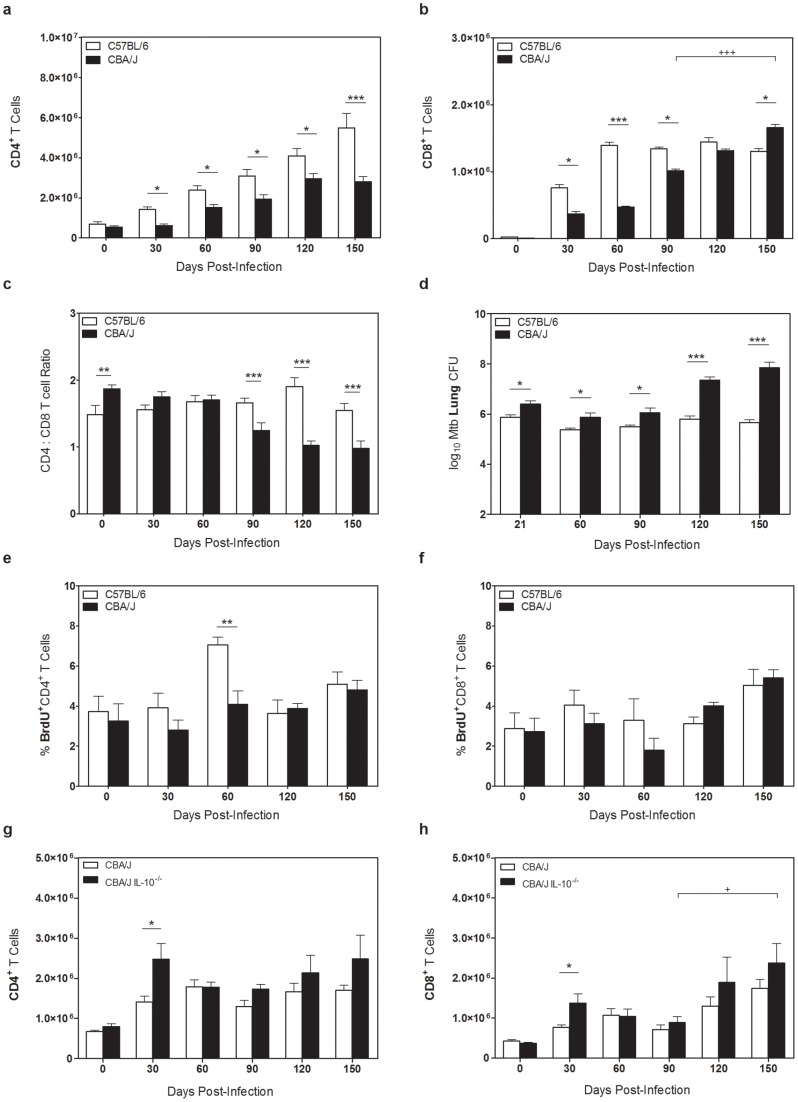
Accumulation and characterization of CBA/J CD8^+^ T cells. C57BL/6, CBA/J, and CBA/J IL-10^−/−^ mice were infected with an aerosolized dose of *Mtb*, and at various times post-infection lungs were removed. (**a, b**) CBA/J and C57BL/6 lung cell were analyzed by flow cytometry for CD4^+^ and CD8^+^ T cells. (**c**) Ratio of CBA/J CD4^+^ to CD8^+^ T cells representative of four independent experiments with 5 mice per group, per timepoint. (**d**) C57BL/6 and CBA/J lungs were homogenized and plated on 7H11 plates for CFU enumeration. (**e, f**) 24 hr prior to necropsy mice were injected with BrdU, and lung cells were analyzed for expression of BrdU^+^ CD4^+^ or CD8^+^ T cells. (**g, h**) Absolute numbers of CD4^+^ or CD8^+^ T cells in wild-type or IL-10^−/−^ CBA/J mice as determined by flow cytometry. Results representative of at least three independent experiments with 5 mice per group, per timepoint. * p<0.05, ** p<0.01, *** p<0.001 as obtained by Student's *t* test. (**b, h**) ^+^ p<0.05, ^++^ p<0.01, ^+++^ p<0.001 as obtained by two-way analysis of variance comparing day 90 to day 150 post-infection.

Following aerogenic infection of CBA/J and C57BL/6 mice with *Mtb* we observed a gradual accumulation of CD4^+^ T cells in the lungs of C57BL/6 mice and significantly fewer CD4^+^ T cells within the lungs of CBA/J mice (**Fig. 1a**), as we have previously described [3], [4]. In contrast to the reduced number of CD4^+^ T cells, CBA/J mice demonstrated a significant late accumulation of CD8^+^ T cells within the lungs as *Mtb* infection progressed (**Fig. 1b**), eventually reaching or surpassing the number of pulmonary CD8^+^ T cells observed in C57BL/6 mice. This late accumulation was absent from C57BL/6 mice which reached a plateau at day 60. Skewing of T cell subset proportions in CBA/J mice could be better appreciated by determination of CD4∶CD8 ratios throughout the course of infection (**Fig. 1c**), where a significant decline in the ratio of CD4^+^ to CD8^+^ T cells was observed by day 90 of *Mtb* infection, preceding the increasing CFU within the lungs of CBA/J mice (**Fig. 1d**) [4], [6]. BrdU staining of pulmonary CD4^+^ (**Fig. 1e**) and CD8^+^ T (**Fig. 1f**) cells from CBA/J mice was not dramatically altered throughout infection, suggesting that the increased numbers of CD8^+^ T cells that were evident in CBA/J mice may not be due to local proliferation, but a consequence of enhanced cellular recruitment. CBA/J mice are known to produce abundant IL-10 in their lungs as *Mtb* infection progresses [5] and IL-10 can stimulate the proliferation of CD8^+^ T cells [41]. Therefore, we examined the numbers of CD4^+^ and CD8^+^ T cells present in the lungs of CBA/J IL-10^−/−^ mice over the course of *Mtb* infection (**Fig. 1g, h**) and did not observe any differences between wild-type and IL-10^−/−^ CBA/J mice in CD8^+^ T cell accumulations at late stages of infection, demonstrating that CD8^+^ T cell expansions occur independent of IL-10. A significant increase in CD4^+^ and CD8^+^ T cells was observed in CBA/J IL-10^−/−^ mice at day 30, reflecting enhanced T_H_1 immunity in these mice (unpublished observations). These data demonstrate that the accumulation of CD8^+^ T cells in the lungs of CBA/J mice during chronic infection is not mediated by IL-10.

### CD8^+^ T cell expansions from *Mtb*-infected CBA/J mice do not align with IFN-γ producing capacity

**Figure 2:**
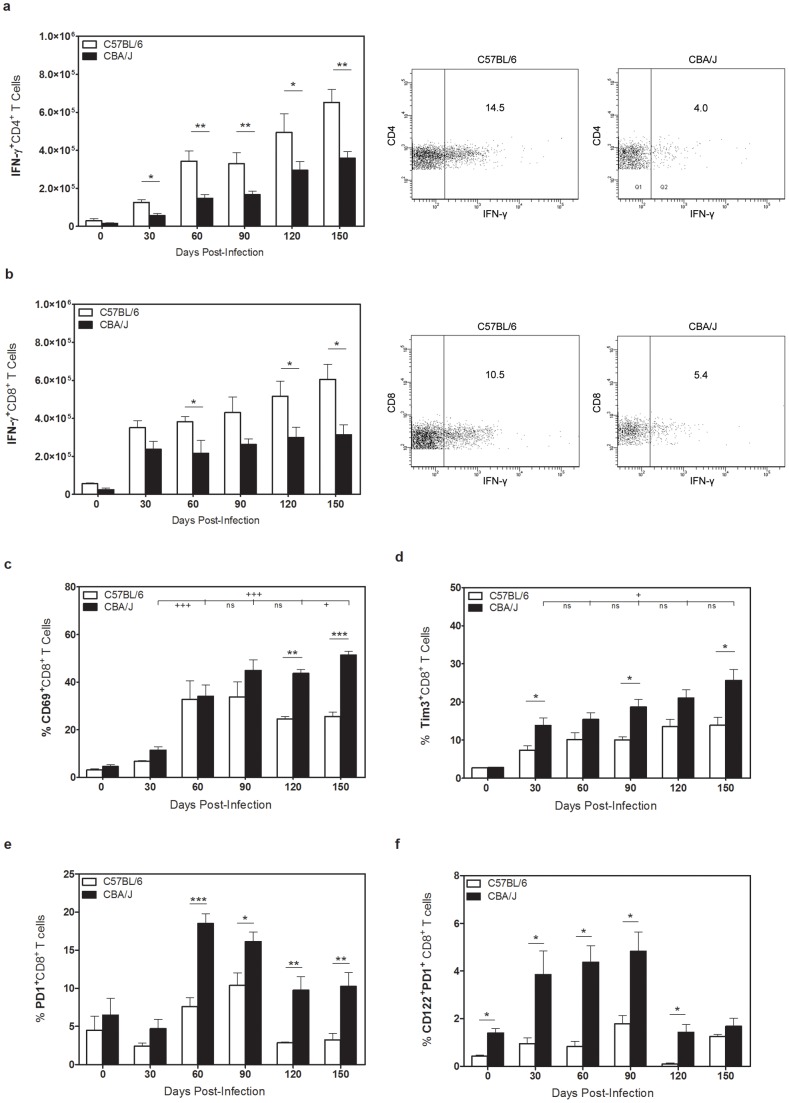
Surface phenotype of pulmonary T cells in CBA/J and C57BL/6 mice. C57BL/6 and CBA/J mice were infected with an aerosolized dose of *Mtb* and at various timepoints post-infection lungs were removed and processed for flow cytometry. Absolute numbers of IFN-γ^+^ CD4^+^ (**a**) or CD8^+^ (**b**) T cells after 4 hr *ex vivo* stimulation with anti-CD3/CD28/GolgiSTOP, with representative flow plots at day 150 post-infection. Absolute numbers of CD8^+^ T cells expressing CD69 (**c**), Tim3 (**d**), or PD-1 (**e**) after *Mtb* infection. (**f**) Absolute number of CD8^+^ T cells expressing both PD-1 and CD122. Data representative of at least two independent experiments with 4 mice per group per timepoint. * p<0.05, ** p<0.01, *** p<0.001 as obtained by Student's *t* test. (**c, d**) ^+^ p<0.05, ^++^ p<0.01, ^+++^ p<0.001 obtained by two-way analysis of variance comparing only CBA/J mice across all timepoints.

We examined the number of T cells that were capable of secreting IFN-γ after *ex vivo* TcR stimulation. As expected, C57BL/6 mice had increasing numbers of CD4^+^ [4] and CD8^+^ T cells that could produce IFN-γ as infection progressed (**Fig. 2a**), which paralleled the increasing numbers of T cells within the lung (**Fig. 1**). CBA/J showed an equivalent increase in IFN-γ-producing CD4^+^ T cells (**Fig. 2b**), albeit at a significantly lower number compared to C57BL/6, as we have previously described [3], [4]. Where the data differed, however, was in the finding that the number of IFN-γ-producing CD8^+^ T cells reached a plateau as early as day 30 post-infection and did not increase any further, despite a significant accumulation of CD8^+^ T cells within the lungs of CBA/J mice. As *Mtb* infection progressed we also observed a significant increase in the number of CD8^+^ T cells from CBA/J mice that could express CD69 (**Fig. 2c**), with over 40% of all CD8^+^ T cells in the lungs of CBA/J mice being activated during chronic infection. CD8^+^ T cells also expressed the suppressor of T_H_1 responses Tim-3 (**Fig. 2d**), PD-1 (**Fig. 2e**), with a small population expressing both PD-1 and CD122 (**Fig. 2f**). Therefore, although highly activated, a proportion of CD8^+^ T cell expansions from chronic *Mtb* infected CBA/J mice had a phenotype that was not associated with T_H_1 cytokine secretion but was instead linked to immuno-suppressive properties including IL-10 production [34]. Because IL-10 production has been closely linked to *Mtb* susceptibility of CBA/J mice [5], we next examined the capacity of CD8^+^ T cells to produce IL-10.

### CD8^+^ T cells from *Mtb*-infected CBA/J mice are capable of secreting IL-10

**Figure 3:**
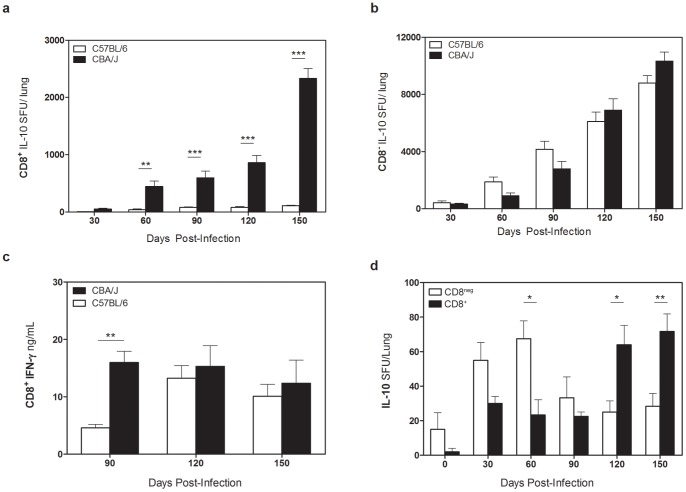
IL-10 production by CD8^+^ T cells from CBA/J mice. C57BL/6 and CBA/J mice were infected with an aerosolized dose of *Mtb* and at various times post-infection lungs were removed and cell populations were purified. Spot-forming units (SFU) representing the absolute number of IL-10^+^CD8^+^ T cells (**a**) or CD8^neg^ T cells (**b**) per lung after 72 hr culture with anti-CD3/CD28. (**c**) Supernatants from (a) were analyzed for IFN-γ levels by ELISA. (**d**) SFU per lung of CBA/J IL-10^+^ CD8^+^ or CD8^neg^ T cells cultured with *Mtb*-infected BMDCs for 72 hr. Data representative of two independent experiments with 4 mice per group per timepoint, * p<0.05, ** p<0.01, *** p<0.001 as obtained by Student's *t* test.

CD8^+^ and CD8^neg^ cells were purified from the lungs of *Mtb*-infected CBA/J and C57BL/6 mice and cultured in IL-10 ELISpot plates for 72 hours with autologous bone marrow-derived dendritic cells (BMDCs) that had been infected with *Mtb* for 24 hours, in the presence or absence of anti-CD3 and anti-CD28. CD8^+^ cells from CBA/J mice, and not C57BL/6 mice, were capable of secreting IL-10 in response to TcR cross-linking (**Fig. 3a**). In contrast, CD8^neg^ cells from both mouse strains were capable of secreting IL-10 under these same conditions (**Fig. 3b**). IL-10-secreting CD8^neg^ cells were predominantly CD4^+^ as adherent cells were removed during cell purification and we have failed to detect IL-10 within B cells and neutrophils in both mouse strains (not shown). We also observed that CD8^+^ T cells from CBA/J and C57BL/6 mice were capable of secreting IFN-γ under the same culture conditions (measured in the IL-10 ELISPOT supernatants) (**Fig. 3c**). Culture of purified CD8^+^ and CD8^neg^ cells from *Mtb* infected CBA/J mice with *Mtb*-infected BMDCs in the absence of TcR cross-linking, indicative of an *Mtb*-specific response, resulted in the secretion of IL-10 from both CD8^neg^ and CD8^+^ cells (**Fig. 3d**). Interestingly, the capacity of CD8^+^ T cells to produce IL-10 increased over time, in parallel to the increasing CFU and CD8^+^ T cell numbers in the lung. The low SFU, relative to TcR cross-linking likely reflects the challenges of delivering of *Mtb* antigen into the appropriate processing pathway for presentation to CD8^+^ T cells.

### CD8^+^ T cells from CBA/J mice are clonally expanded

**Figure 4:**
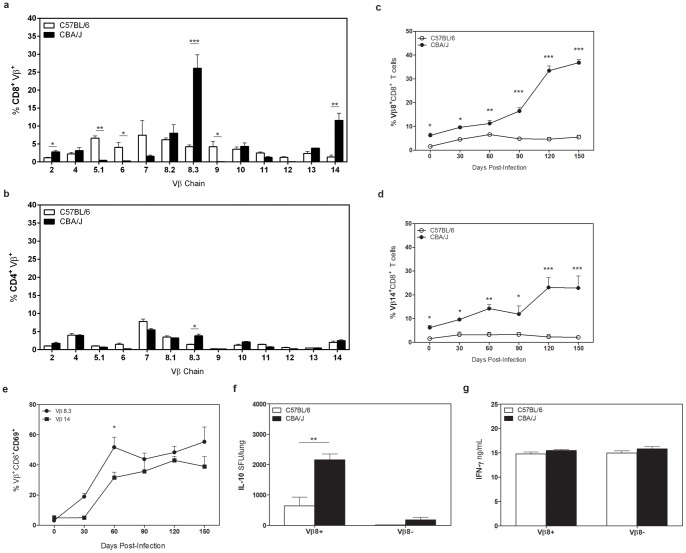
Vβ TcR expression in CBA/J and C57BL/6 mice. C57BL/6 and CBA/J mice were infected with an aerosol dose of *Mtb* and at various timepoints post-infection lungs were removed and processed for flow cytometry. Percentages of CD8^+^ (**a**) or CD4^+^ (**b**) T cells expressing specific Vβ TcRs at day 120 post-infection. Percentages of CD8^+^ T cells expressing Vβ8 (**c**) or Vβ14 (**d**) TcR over the course of *Mtb* infection. (**e**) Percentages of Vβ8^+^ or Vβ14^+^ CD69^+^CD8^+^ pulmonary T cells over time. (**f**) Vβ8^+^ and Vβ8^neg^ T cells from day 120 post-infection were cultured for 72 hr with anti-CD3/CD28 and IL-10 SFU determined by ELISpot. (**g**) Supernatants from (a) were analyzed for IFN-γ levels by ELISA. Data representative of at least two independent experiments with 4 mice per group per timepoint. * p<0.05, ** p<0.01, *** p<0.001 as obtained by Student's *t* test.

We determined the clonal repertoire of the CD8^+^ T cells that accumulated in the lungs of CBA/J mice during *Mtb* infection to determine whether the reactivity of CD8^+^ T cells was potentially driven by a dominant antigen, a concept supported by the finding of T cell clonal expansions in TB patients [42], [43]. At late stages of *Mtb* infection, we observed that CD8^+^ T cells from CBA/J mice primarily expressed two variable regions of the TcR beta chain (Vβ). Vβ8 (8.2,8.3) and Vβ14 were expressed by approximately 45% of all CD8^+^ T cells in CBA/J mice at day 120 after *Mtb* infection, compared to a less restrictive repertoire in C57BL/6 mice (**Fig. 4a**). Vβ expression was comparable on CD4^+^ T cells from both mouse strains (**Fig. 4b**), indicating a unique expansion within the CD8^+^ T cell pool in CBA/J mice as *Mtb* infection progressed. More detailed examination over the course of *Mtb* infection showed that the percentage of CD8^+^ T cells expressing Vβ8 or Vβ14 was always significantly higher throughout *Mtb* infection in CBA/J mice, and this population significantly expanded further at late time points of *Mtb* infection (**Fig. 4c, d**). Similar to our findings with CD8^+^ T cell expansions, we found that both Vβ8 and Vβ14 CD8^+^ T cells were highly activated (**Fig. 4e**).

We determined the IL-10 producing capacity of CD8^+^ Vβ8^+^ T cells using magnetic bead separation, which purified the dominant Vβ8 expressing CD8^+^ population. CD8^+^ Vβ8^+^ and CD8^+^ Vβ8^neg^ T cells were isolated from *Mtb*-infected CBA/J mice or C57BL/6 mice (controls) at day 150 post *Mtb* infection and cultured in IL-10 ELISpot plates with autologous IL-10 deficient BMDCs in the presence of anti-CD3/CD28. Significantly more CD8^+^ Vβ8^+^ T cells from *Mtb*-infected CBA/J mice were capable of producing IL-10 than CD8^+^ Vβ8^neg^ T cells from CBA/J mice or from CD8^+^ Vβ8^+^ and CD8^+^ Vβ8^neg^ T cells from C57BL/6 mice (**Fig. 4f**). These data indicate a dominant role for CD8^+^ Vβ8^+^ expressing T cells in the IL-10 production we previously observed in purified CD8^+^ T cell cultures. Culture supernatants were also assayed for IFN-γ by ELISA (**Fig. 4g**) and our data indicate that CD8^+^ Vβ8^+^ T cells can have dual secretion of IL-10 and IFN-γ, or represent a mixed population, as has been described by others [44].

### *In vivo* depletion of IL-10 producing CD8^+^ T cells during chronic *Mtb* infection alters pro-inflammatory responses but fails to modify the bacterial load

**Figure 5:**
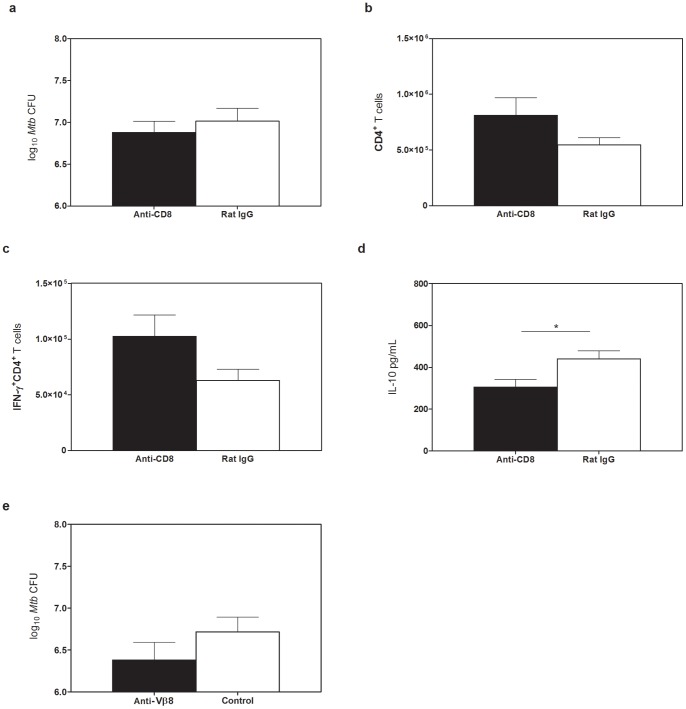
CD8^+^ T cell depletion in CBA/J mice. CBA/J mice were infected with an aerosolized dose of *Mtb* and from day 90–120 were treated weekly with depletion antibody via intraperitoneal injection then sacrificed at day 125 post-infection. (**a**) Lungs of anti-CD8^+^ T cell depleted or control mice were homogenized and plated on 7H11 plates and CFU enumerated after 21 days. Absolute number of total CD4^+^ T cells (**b**) or IFN-γ^+^CD4^+^ T cells (**c**) after CD8^+^ T cell depletion as determined by flow cytometry. (**d**) Levels of pulmonary IL-10 after CD8^+^ T cell depletion as determined by ELISA. (**e**) Lungs of anti-Vβ8 depleted or control mice were homogenized and plated onto 7H11 plates and CFU enumerated. Control group  =  no treatment and isotype control. Results representative of at least two independent experiments with 5–10 mice per group. Depletion resulted in 95% reduction in cell number, as determined by flow cytometry. * p<0.05, ** p<0.01, *** p<0.001 as obtained by Student's *t* test.

CD8^+^ T cells or Vβ8^+^ cells were depleted from wild-type CBA/J mice from day 90–120 after *Mtb* infection, a time when IL-10 and Vβ8^+^ CD8^+^ T cells were increasing in the lungs of *Mtb*-infected CBA/J mice [5]. Following CD8^+^ depletion, *Mtb* CFU (**Fig. 5a**), total CD4^+^ T cell numbers (**Fig. 5b**) and IFN-γ-producing CD4^+^ T cells (**Fig. 5c**) were all moderately altered but data did not reach statistical significance. Interestingly, CD8^+^ T cell depletion led to a significant decrease in the total amount of IL-10 in the lungs of CBA/J mice (**Fig. 5d**), reflecting the IL-10-secreting capacity of CD8^+^ T cells we observed *in vitro*. Depletion of CD4^+^ T cells led to significantly increased *Mtb* burden and mortality before day 120 (not shown) showing that depletion of a known protective T cell subset increased susceptibility. Specific depletion of Vβ8^+^ cells also failed to significantly impact the *Mtb* burden (**Fig. 5e**), although a modest reduction in CFU was similarly observed. This modest reduction, similar to our findings with CD8^+^ T cell depletion, suggests that although IL-10 producing CD8^+^ T cells may not negatively impact the growth of *Mtb* during the timeframe we investigated, they do not provide protection.

## Discussion

We have demonstrated that clonal expansions of CD8^+^ T cells from CBA/J mice accumulate in the lung over the course of *Mtb* infection. This accumulation did not yield an equivalent increase in IFN-γ^+^CD8^+^ T cells and, upon further phenotypic examination, we discovered that highly activated CD8^+^ T cells from CBA/J mice expressed the T cell dysfunction markers PD-1 and Tim-3, as well as co-expression of PD-1 and CD122 suggesting possible immunosuppressive activity. After *ex vivo* purification and culture, it was shown that CD8^+^ T cells from CBA/J mice were capable of secreting IL-10 after TcR stimulation and in response to *Mtb* infected BMDCs. Depletion of Vβ8^+^ cells or the entire CD8^+^ T cell population in *Mtb*-infected CBA/J mice led to a significant reduction in pulmonary IL-10 levels, however, we observed no significant change in pulmonary CFU.

Using an *Mtb*-susceptible mouse strain, we reveal that CD8^+^ T cells that are capable of producing IL-10 can accumulate within the lung during *Mtb* infection. CD8^+^ T cells within the lung expressed CD69, indicative of activation and functional capacity, yet failed to show a concomitant enhancement of IFN-γ-producing capacity. These findings indicate that highly activated CD8^+^ T cells within the lung have alternate function, which we show here to be the capacity to secrete IL-10, measured by ELISPOT due to the known difficulties of measuring IL-10 by intracellular flow cytometry. Altered function was associated with the co-expression of a variety of receptors know for negative regulation of cell function (PD-1, CD122, Tim-3) [25], [28], [33], [34]. In support of our findings, previous studies have shown that in chronic murine *Mtb* infection PD-1^+^ T cells can proliferate but fail to secrete IFN-γ unless this inhibition is overcome by direct TcR stimulation [45], [46]. It is unclear at this time why CD8^+^ T cells with inhibitory properties arise in CBA/J mice as *Mtb* infection progresses but we can hypothesize that this is a consequence of enhanced immune activation and subsequent exhaustion due to increasing bacterial loads in this mouse strain (Fig. 1) [4], [6].

Our failure to observe any significant change in CFU following CD8^+^ or Vβ8^+^ T cell depletion can be interpreted in several ways, the simplest being that IL-10-producing CD8^+^ T cells have no biological influence on the control of *Mtb* infection. While this is a possibility, we would reason that at the least the presence of IL-10-producing CD8^+^ T cells can be a putative biomarker of TB disease progression as they are only observed in a mouse strain of *Mtb* susceptibility, and IL-10 producing T cells have been previously described in the blood of TB patients [37]. In support of a negative role for CD8^+^ T cells in CBA/J mice, *in vivo* depletion led to a moderate, albeit not significant, decrease in CFU. Thus is in contrast to CD4^+^ T cell depletion in which all the mice were either dead or moribund before the necropsy time point (data not shown). These findings indicate that some CD8^+^ T cells from CBA/J mice fail to contribute to protection in a similar manner than might be expected from studies of other mouse strains [47]–[49]. One possibility for our failure to detect significant changes in CFU following CD8^+^ T cell depletion is our observation that CD8^+^ T cells can secrete both IL-10 and IFN-γ. Depletion of all CD8^+^ T cells would also remove a protective population, leading to a neutral effect. Previous studies from our laboratory have shown that blocking the action of IL-10 in CBA/J mice during *Mtb* infection provides enhanced protection [5], and we consider that blocking the action of IL-10 from IL-10/IFN-γ-secreting CD8^+^ T cells contributed to this phenotype.

In addition to IL-10 production, we also observed that CD8^+^ T cells from CBA/J mice had severely restricted diversity of their TcR repertoire which would significantly limit the breadth of antigens that CBA/J CD8^+^ T cells can recognize. CBA/J mice have an endogenous mouse mammary tumor virus (MTV-6) that selectively deletes various TcR Vβ chains such as Vβ8.1, and Vβ 17a [50], [51] however this alone is not sufficient to explain the limited TcR diversity in CBA/J mice. It is possible that *Mtb* infection leads to specific deletion of certain subsets of protective CD8^+^ T cells or is driving clonal expansion of less protective cells (Vβ8, Vβ14). Alternatively Vβ8/Vβ14 CD8^+^ T cells in CBA/J mice may respond to a dominant *Mtb* antigen(s) expressed *in vivo*, where persistent responsiveness leads to immune exhaustion and subsequent regulation through receptors such as PD-1 and Tim-3. Clonal CD8^+^ expansions have been described in TB patients [52], including pediatric TB [42], supporting the relevance of our findings to TB in man. Clonal CD8^+^ expansions are also common in the elderly and in many viral models [42], [43], [53]–[58], yet their importance in *Mtb* infection is still unclear.

In summary, we show using an *Mtb*-susceptible mouse strain (CBA/J) that clonal expansions of CD8^+^ T cells with the capacity to produce IL-10 can arise during chronic *Mtb* infection. In combination with our previous finding that blocking the action of IL-10 can improve outcome of *Mtb* infection [5], these data further support a negative role for IL-10 in the generation and maintenance of protective immunity against *Mtb* infection. Our findings are of significance because vaccines that specifically stimulate CD8^+^ T cells are currently under development [59]–[63]. Given that IL-10-secreting CD8^+^ T cells with the potential to negatively impact control of *Mtb* infection can naturally arise, generating such cells in response to vaccination should be considered.
